# Editorial: Theories, methods, practices, and fields of digital social research

**DOI:** 10.3389/fsoc.2024.1437401

**Published:** 2024-06-28

**Authors:** Felice Addeo, Angela Delli Paoli, Gabriella Punziano

**Affiliations:** ^1^Department of Politic and Communication Sciences, University of Salerno, Fisciano, Campania, Italy; ^2^Department of Humanities, Philosophy and Education, University of Salerno, Fisciano, Campania, Italy; ^3^Department of Social Sciences, University of Naples – Federico II, Naples, Italy

**Keywords:** digital methods, editorial, digital social research, digital society, social research methods, digital sociology

The digital nuances of contemporary societies are becoming thicker and thicker as technology is progressively pervading every aspect of our social life; just think of the way in which many, if not most, social actions and social relationships, both formal and informal, are mediated and experienced through digital devices. This occurrence has an impact on the very concept of digital social research, which, according to some authors, might even be a redundant expression. In fact, as Pink (2019) wittily pointed out, because the digital, social, and material aspects of our worlds are now inextricably linked, social research is currently almost always digital in both its subject matter and methodology, as our research strategies and interactions are, even if only indirectly, connected to digital platforms, and practices.

Obviously, the theoretical question of new social formations, phenomena, and practices arising through internet access is different and separated from the question of methods to carry out social research using ICT (Information and communication technology). However, these two themes co-occur and need to converge in the recognition of the digital not only as a topic of social research but especially as a way of transforming social research both in terms of topics and in terms of methods.

Even though there is still no solid set of shared definitions and concepts in the social science community regarding the social study of the digital, in our opinion it is worthwhile to consider digital social research as a disciplinary field in its own right. The swirling yet gradual evolution of digital technologies is causing, among other consequences, a constant and potentially unlimited production of information on every human practice and activity experienced through the Internet.

The last decade has seen deep changes in the way people use new Internet-related technologies to manage their private data. People have moved from secretly exchanging small amounts of anonymised data to sharing huge volumes of personal data that can be traced at any time, thus blurring the boundaries between what is public and what is private. This data is commonly defined as “Digital traces,” i.e., the footprints we leave behind on a daily basis by surfing the Internet, acting and interacting online with other people or with social networking platforms (Hinds and Joinson, 2018; Keusch and Kreuter, 2022). Digital traces are a heterogeneous set: from the information we share on social platforms (likes, comments, tweets, etc.) to the websites we visit or the products we search for on the ecommerce platforms. These types of information are also recognized as “Big Data,” and, according to some scholars (Lewis, 2015; Molteni and Airoldi, 2018), are naturalistic data, being them “found” and spontaneously generated by users and not requested or provoked by researchers. According to Kitchin, this epistemological vision of Big Data applied to the social sciences act as a discursive rhetorical device orienting research practices toward a mere empiricism in which theories progressively lose their relevance (Kitchin, 2014) in favor of data. Similarly, Fuchs (2019) believes that a digital positivism has emerged from this view, which risks influencing digital social research with the idea that theoretical reflection is no more than a mere ornament, reduced to a sterile list of superficial definitions of key concepts. However, this alleged total shift from theory-driven to data-driven knowledge making is not sustainable from a theoretical and methodological point of view. On the one hand, digital social research must not be reduced to a branch of data science, on the other hand, of course, it cannot ignore it either (Veltri, 2019), but must look at data from a critical perspective. Every social scientist knows that ‘data' is only such if there is a conceptual framework in which it is collected, analyzed and interpreted (De Martino et al., 2020, 2021). The likes, the comments, the tweets, the click views, are not inherently meaningful. Data are analyzed through specific lenses that influence their interpretation. Even the algorithms used to collect and analyse digital data are intrinsically linked to a specific theory and/or method (Giuffrida et al., 2016). Therefore, digital social research should pay more attention to a systematic and critical application of social theories and ethics when it deals with the study of digital society and its peculiar phenomena, dynamics, and practices (Fuchs, 2019).

We could say that digital social research instead that a new empirism calls for hermeneutics and interpretation at different levels of the research process. At the level of goal setting, the ever-changing nature of digital society and the impact of the digital on mainstream sociological concepts such as identity, community, relationships, and capitals and so on, imposes appropriate research questions.

Research questions are crucial in social research in general, both digital and not. Without them, the collection of information is impossible or meaningless since everything would appear important (Miles and Huberman, 1984). Thus, digital social research cannot be limited to a mere analytic process, a purely computational and data analysis approach. Also at the level of data collection and due to the non-neutrality of algorithms the role of human interpretation becomes significant also in data mining for example in selecting attributes, features and categories for data collection. At the level of data analysis, technological interpretation of affordances (the socio-technical architectures of media such as likes, tags, shares, and hashtags) and their role in structuring the digital actions and interactions is essential for making sense of the results. Although the interpretive role is more evident in qualitative analysis such as narrative analysis or digital ethnography and it involves making sense of intertextual, trans-medial, multimodal and interdiscursive narratives, it is also crucial in computational analysis where the equivalence between correlation and causation is frequent. Big data based correlation is not sufficient to understand social phenomena (correlation is not causation) (Delli Paoli and Masullo, 2022).

The digital society is an ever-changing and rapidly evolving research object; therefore, there is the need for theoretical and methodological frameworks that may help us to understand how new technologies interact with people in the social daily life. At first, when the majority of social scientists were convinced that studying digital practices no longer meant moving away from reality, the social sciences made an effort to adapt traditional methods to research digital contexts. Subsequently, this effort was aimed at developing new methodological tools designed specifically to study the web.

However, neither reworking traditional techniques nor the development of new digital tools could be considered the only valid and reliable way to do digital social research. In the former case, traditional social research methods and techniques may prove to be unsuitable for the study of certain digital practices or contexts (e.g., the study of online communities or social media-related phenomena). However, new methodological tools, with a clear digital nature, may often turn out to be extemporaneous attempts, destined to become obsolete in a very short period of time (Addeo and D'Auria, 2022). Moreover, digital social research frameworks should take into account the possibility that new technologies not only change, even radically, during our experience with them, but that over time the ways in which they 'intelligently' interact with us, learn from or with us in the course of our dealings with them, and make decisions will increase and intensify (Pink, 2019).

At the current stage of the epistemological development of the social sciences, it is difficult to find conceptual and operational definitions of the key concepts in the digital social research field that are shared by the majority of the social science scientific community. This is not necessarily a bad thing for social science, if we consider that this *in fieri* state of the art could pave the way for challenging digital positivism while promoting critical digital research practices. Social scientists should 'only' be fully aware that the knowledge drawn from the use of digital and all the web-related technologies is always fuzzy, revisable and highly prone to obsolescence due to the continuous flourishing of online social practices and the creative ways in which individuals' online activities are embedded in data. Digital social research should therefore also be critical, marked by transdisciplinarity and intersectionality; it should aim to understand, and eventually interiorise, how digital technologies are conceptualized and studied in other disciplines and outside academia. Digital social researchers should conceptualize but also and above all practice the processes through which technology is designed, understood, and implemented in social life (Fuchs, 2019; Pink, 2019).

This research topic explores the challenges and advantages as well as the pitfalls and problems of the digital, conceived here both as an object of research and as a methodological tool, and offers epistemological and methodological insights and examples of what it means to do digital social research. The Research Topic collects articles from academics and scholars belonging to different research fields (e.g., sociology, education, and political science) conducting innovative research on several compelling social science Research Topics, which demonstrates both the increasing relevance of digital in daily life as well as the use of digital media tools to address social research questions. The essays collected are significant examples of empirical social science research performed in the digital era, through a wide range of methodological approaches, some original and others more traditional, in the context of digital technology.

The digital society is constantly changing, in line with the rapid evolution of technologies that are redefining its practices. Unfortunately, the spread of technology does not travel at the same speed both between different countries and within the same country, and this is mainly due to the so-called digital divide, understood as inequality in access to and use of new digital technologies (Hilbert, 2015; Van Deursen and Van Dijk, 2019). Drawing from research that have successfully measured the Digital Capital in UK (Ragnedda et al., 2019), Addeo et al. propose a research path to detect and validate this concept in the Italian context. The results show that the operationalisation of Digital Capital works also in Italy, thus legitimizing the idea it could be conceived as an independent capital. Laskar offers an in-depth analysis of the digital divide in India, showing how socio-economic factors, especially urban-rural differences, are a key determinant of digital inequalities.

The emergence of new digital entities, such as the metaverse, and the risks associated with them require an effort of understanding that Pascali fully succeeds in making, highlighting the urgency of rethinking traditional forms of preventive and repressive measures to counter deviant and illegal drifts in digital spaces. A well-researched digital practice on which there is no agreement by the scientific community is online propaganda. Nerino effectively proposes the use of Druckman's Generalizing Persuasion Framework to address this gap, thereby emphasizing the role of cultural and cognitive sociology. Health is one of the sectors that is benefiting most from the digital revolution. Lenzi and Iazzetta disclose how the use of social media could increase knowledge about diabetes and obesity, suggesting and motivating targeted public health strategies.

The relevance of the Health field for the development of digital social research is also evident from the fact that the digitisation of society has undoubtedly been accelerated by the pandemic crisis. This process had a huge epistemological and methodological impact on the social research: COVID-19 upturned the social research inertia as regard digital methodological innovations (Velotti et al., 2021). The problem caused by the pandemic crisis are still being experienced in all sectors from Health to Policy, from Economy to Education; COVID-19 aftermath will be felt for a long time to come. That it is why several papers in this Research Topic deals directly or indirectly with COVID-19 related subjects.

In Italy, one of the strongest consequences of the pandemic has been the acceleration of distance learning practices by schools and universities. Two papers are dedicated to this Research Topic: Lo Presti provides an argued assessment of the social impact of the Distance Learning (DaD) within the framework of the Positive Thinking Evaluation; Faggiano and Fasanella reflect on future scenarios for learning offered not only by common Distance Learning tools but also by the adoption of VR in educational contexts.

The COVID-19 pandemic has also exacerbated the issue of continuous infodemic of fake news and (mis)information to such an extent that it has become a major cause of public concern; not least because misinformation has been both cause and effect of institutional mismanagement of the pandemic crisis (Ruiu, 2021). One of the piece de resistance of disinformation practices has been the vaccination campaign. Murero wittily discusses how the manipulation tactic called Coordinated inauthentic behavior (CIB), using a mix of authentic, fake and duplicate accounts on social media, massively misled the online debate on COVID-19 vaccination. Pilati et al. analyse, from a worldwide perspective, the relationship between the Infodemic Risk Index and the epidemic wave, finding a decrease in misinformation on Twitter as the number of COVID-19 confirmed cases increases. During the pandemic crisis, Twitter became a kind of “official” social media used by health and government institutions to disseminate information on COVID. Gozzo and D'Agata explore how Twitter helped build a digital community based on a shared digital culture that contributed to the spread of ontological forms of security. Taddei et al. examine the contributions of digital social research to develop E-Health and Telemedicine in Southern Italy in a post-COVID-19 scenario, identifying those issues that need to be addressed in order to reduce the existing gaps and inequalities. As highlighted by some authors (Velotti et al., 2021) COVID-19 crisis paradoxically has been a noteworthy prospect for social science study to pursue innovative methodology. Vaccaro et al. illustrate the strengths and the weakness of a qualitative method, the SONAR-global Vulnerability/Resilience Assessment, for defining and analyzing vulnerabilities during the COVID-19 pandemics. An innovative spatial analysis methods was developed and implemented by Lenzi and Truglia in order to analyse the territorial spillover of COVID-19 infections in Rome proving how useful digital methods could be when studying rapidly changing phenomenon as the spread of a viral infection on an urban scale. Using a combination of traditional (Factor Analys and Cluster Analysis) and innovative (Topic Modeling) techniques, Acampa et al. investigate the narratives on the pandemic and vaccines on social media platform.

Digital social research requires a robust and epistemologically grounded methodological apparatus, which is why many contributions in this Research Topic aim at addressing methodological questions. Drawing on this, the paper from De-Groot et al. is of great relevance as it fills an important gap in current scientific research: by combining web-analytics with quantitative and qualitative research methods, it develops a ground-breaking framework for monitoring the citizen science landscape, the CS Track. On the same wavelength, Martini discusses from a sociological perspective the advantages of adopting a quintuple helix model to predict possible future digital scenarios and their consequences from economic, social, and technological perspectives. Poliandri et al. critically review different approaches to conducting online focus groups, subsequently porpoising an online focus group protocol, used as part of a research project carried out Italy, that overcomes the limitations previously highlighted. In their study about the Italian *digital diaspora* in China, Moffa and Di Gregorio offer a timely methodological account of the advantages and disadvantages of using messaging and social media apps as tools for qualitative research. Starting from sound methodological premises, Caroleo et al. assess the impact of SEO techniques on the way information on political issues circulates and influences public debate and opinion.

The Cambridge Analytica cased a data policy shift, the so-called “APIcalypse,” that dramatically shaped the digital research methods, greatly limiting social researchers' access to digital data. Based on the results of a survey on Italian researchers, Trezza critically reflects on the way these restrictions have altered, positively but mostly negatively, current social research practices and suggests social research to make a self-reflexive effort to diversify research platforms and to act ethically with user data.

La Rocca and Boccia Artieri a offer two valuable contributions reflecting on the use of hashtags in social research: the first paper provides a thorough review of this area of research, outlining the features of what can be called hashtag research. The second contribution develops an innovative interpretive proposal of the hashtag as a relational social form, thus formalizing a model to analyze the changeable meaning of the hashtags (La Rocca and Boccia Artieri b).

One of the methodological approaches that has been able to adapt best to the digital revolution has been the ethnographic approach, and two papers in this Research Topic reflect excellently on its current developments. Padricelli and Punziano starting with an overview of the evolution of ethnographic studies in the social sciences, propose a conceptual analysis that traces the main pillars of the current development of the entnographic method and identifies its possible future directions. Masullo and Coppola focus specifically on the digital evolution of the ethnographic method, offering a careful examination of the advantages and disadvantages of this method through a practical case study of a web community of Italian asexual people.

## Author contributions

FA: Conceptualization, Methodology, Project administration, Supervision, Validation, Writing – original draft, Writing – review & editing. AD: Conceptualization, Methodology, Project administration, Supervision, Validation, Writing – original draft, Writing – review & editing. GP: Conceptualization, Investigation, Methodology, Project administration, Validation, Writing – original draft, Writing – review & editing.
